# Randomized placebo control study of insulin sensitizers (Metformin and Pioglitazone) in psoriasis patients with metabolic syndrome (Topical Treatment Cohort)

**DOI:** 10.1186/s12895-016-0049-y

**Published:** 2016-08-17

**Authors:** Surjit Singh, Anil Bhansali

**Affiliations:** 1 Department of Pharmacology, All India Institute of Medical Sciences (AIIMS), Jodhpur, 342005 India; 2 Department of Endocrinology, Post Graduate Institute of Medical Education and Research (PGIMER), Chandigarh, 160012 India

**Keywords:** Psoriasis, Metabolic syndrome, Insulin sensitizers, Metformin, Pioglitazone

## Abstract

**Background:**

Increased prevalence of metabolic syndrome (MS) is observed in psoriasis. Metformin has shown improvement in cardiovascular risk factors while pioglitazone demonstrated anti proliferative, anti-inflammatory and anti angiogenic effects. Study objective is to evaluate the efficacy and safety of Insulin sensitizers (metformin and pioglitazone) in psoriasis patients with metabolic syndrome (MS).

**Methods:**

Single centre, parallel group, randomized, study of metformin, pioglitazone and placebo in psoriasis patients with MS.

**Results:**

Statistically significant improvement was observed in Psoriasis Area and Severity Index (PASI), Erythema, Scaling and Induration (ESI) and Physician global assessment (PGA) scores in pioglitazone (*p* values – PASI = 0.001, ESI = 0.002, PGA = 0.008) and metformin groups (*p* values – PASI = 0.001, ESI = 0.016, PGA = 0.012) as compared to placebo. There was statistically significant difference in percentage of patients achieving 75 % reduction in PASI and ESI scores in metformin (*p* value – PASI = 0.001, ESI = 0.001) and pioglitazone groups (*p* vaue – PASI = 0.001, ESI = 0.001). Significant improvement was observed in fasting plasma glucose (FPG) and triglycerides levels in metformin and pioglitazone arms. Significant improvement was noted in weight, BMI, waist circumference, FPG, triglycerides and total cholesterol after 12 weeks of treatment with metformin while pioglitazone showed improvement in FPG, triglyceride levels, systolic blood pressure (SBP), diastolic blood pressure (DBP), total cholesterol and LDL cholesterol levels. There was no difference in pattern of adverse drug reaction in three groups.

**Conclusion:**

Insulin sensitizers have shown improvement in the parameters of MS as well as disease severity in psoriasis patients.

**Trial registration:**

CTRI Registration Number: CTRI/2011/12/002252. Registered on 19/12/2011.

## Background

Psoriasis is a chronic, inflammatory multisystemic disorder with genetic basis affecting 2-3 % of world population and affecting about 0.4 % of Asians [1]. Psoriasis has been found to be associated more commonly with obesity, metabolic syndrome (MS) [1, 2], diabetes mellitus [3] and increased cardiovascular (CVS) mortality and morbidity [4–6]. Metabolic syndrome (MS) is a cluster of risk factors including central obesity, atherogenic dyslipidemia, hypertension and glucose intolerance and is a strong predictor of cardiovascular diseases, diabetes and stroke [7–9]. Many cytokines (e.g. interferon-γ, TNF-α, IL-6**,** IL-8, IL-12, IL-17, IL-19 and IL-23) involved in the pathogenesis of psoriasis are also known to contribute to the cascade of metabolic syndrome such as hypertension, dyslipidemia and insulin resistance [10]. Prodifferentiating, antiproliferative, anti-inflammatory and antiangiogenic effects of Peroxisome proliferator-activated receptor- γ (PPAR-γ) ligands may potentially have beneficial role in psoriasis [11–13] as exemplified by demonstrated efficacy of Thiazolidinediones (TZDs) in treatment of psoriasis [14–18]. Metformin is an ‘insulin sensitiser’, lowers glucose levels without increasing insulin secretion. It has shown additional beneficial effects in adults with type 2 diabetes, including weight reduction, decreasing hyperinsulinemia, improving lipid profiles, augmented fibrinolysis and enhanced endothelial function [19–21], that all are usual metabilic abnormalities observed in subjects with MS. Therfore we anticipated that such pharmacological effects observed with metformin might be of use in psoriatic patients with MS. To the best of our knowledge no study till date has evaluated metformin and pioglitazone head to head in patients of psoriasis with MS. Present study was planned as comparative evaluation of safety & efficay of metformin with pioglitazone in placebo controlled setting in patients of psoriasis with MS.

## Methods

### Clinical trial design

Study was approved by Institute Ethics committee, Post Graduate Institute of Medical Education and Research. This clinical trial was a single centre, parallel group, randomized, open label with blinded endpoint assessment of metformin, pioglitazone and placebo in psoriasis patients with MS satisfying inclusion and exclusion criteria. Our study is a part of larger study in which we evaluated the prevalence of MS in psoriasis. Then psoriasis patients having MS were divided into systemic (moderate to severe psoriasis, randomized into metformin and placebo arms) and topical treatment cohort (mild to moderate psoriasis, randomized into metformin, piolglitazone and placebo arms) and were evaluated for the effect of insulin sensitizers on disease parameters and MS. In this paper, we have discussed the results of topical treatment cohort.

All patients visiting psoriasis clinic at our Institute were screened for MS and other eligibility criteria. Both males and females, > 18 years with plaque psoriasis [mild to moderate disease severity (<10 % of body surface area) [22], on treatment (had taken even a single application of topical therapy in the past) and treatment naïve (no past history of treatment for their disease)] and having MS i.e. the presence of three or more criteria of the modified National Cholesterol Education Program’s Adult Treatment Panel III (NCEP ATP III) [23]: waist circumference > 90 cm in men and > 80 cm in women, hypertriglyceridemia ≥ 150 mg/dl, high density lipoprotein (HDL) cholesterol < 40 mg/dl in males and < 50 mg/dl in females, blood pressure ≥ 130/85 mmHg and fasting plasma glucose ≥ 110 mg/dl and willing to provide written informed consent were included in the study. Patients with severe disease, on topical therapy other than coal tar, pregnant or nursing women, significant hepatic impairment (serum bilirubin, AST, ALT and alkaline phosphatase >1.5 times the upper limit of normal), renal insufficiency - serum creatinine ≥1.5 mg/dL (men) or ≥1.4 mg/dL (women) and contraindication to metformin and pioglitazone were excluded from the study.

Clinical examination including psoriasis area and severity index (PASI) [22] scores and erythema, scaling and induration (ESI) scoring [24] was done. Clinical photographs of patients were taken at baseline and post treatment. Baseline investigations were done and eligible patients were randomized in an open label manner to either placebo (empty gelatin capsules), metformin 1000 mg once daily (O.D) or pioglitazone 30 mg O.D groups for a period of 12 weeks, after taking written informed consent. All patients were given standard topical 5 % coal tar ointment in addition to study drugs. The randomization codes were computer generated. Randomization codes were concealed in an opaque envelope. The drug dispensation was done by a person who was not involved with the assessment of the study endpoints. Evaluation for efficacy parameters was done at 0 and 12 weeks. Safety evaluation was also done throughout the study.

### Efficacy evaluation

Blinded end points assessment of the efficacy parameters was done at 12 weeks. Psoriasis lesions were evaluated using psoriasis area and severity index (PASI) scores and erythema, scaling and induration (ESI) score [24]. Each component of ESI was graded from 0 to 3; 0 – clear, 1 - mild, 2 - moderate, 3 - severe. The most severe condition was given 9 points whereas absence of disease been given 0 points.

Also all the parameters of MS as defined by modified National Cholesterol Education Program’s Adult Treatment Panel III (NCEPIII) criteria [23] were assesed at baseline and 12 weeks. Serum IL-6 and TNF-α levels was done at 0 and 12 weeks in subgroup (10, 7 and 9 patients in placebo, metformin and pioglitazone groups respectively) of patients by Human ELISA kit (RayBiotech, Inc. Georgia. USA).

The primary efficacy end point was mean change in PASI, ESI and PGA scores from baseline after 12 weeks of therapy between three treatment groups given along with standard treatment for psoriasis. The Secondary efficacy end point were number of parameters of MS improved, change in individual parameters of MS, IL -6 and TNF – α from baseline after 12 weeks of treatment with metformin, pioglitazone or placebo. The change in Physician Global Assessment (PGA) from baseline and percentage of patients achieving 75 % reduction in ESI and PGA score in the three treatment groups were other end points.

### Sample size calculation

Assuming a standard deviation of 2 in PASI scores, and a difference of 2 in PASI score between drug and placebo arm at 12 weeks to be clinically significant at α = 0.05 and with 80 % power, a sample size of 16 patients per group has been calculated and with a dropout rate of about 20 %, 19 patients will be required to be included in each group.

### Statistical analysis

Data was expressed as Mean ± SD (95 % confidence intervals), numbers (percentages) and median (interquartile range). Baseline characteristics between three treatment groups were compared using one way ANOVA for numerical variables and Chi-Square test for categorical variables. Analysis was carried out using intention to treat principle.

Mean changes in PASI, ESI and PGA scores at 12 weeks from baseline between three treatment groups were compared using One way ANOVA followed by post hoc Scheffe. Chi-Square test or Fischer’s Exact test was used to compare the categorical variables. Intra group comparison of mean changes in individual parameters of MS and lipid profile was carried out by paired *T*-test and inter group comparison by One way ANOVA. Difference in changes in serum levels of IL-6 and TNF-α between the groups was done by One way ANOVA.

Results were analyzed as Intention-to-treat analysis with last observation carry forward (LOCF). A two-sided *P*-value less than 0.05 was considered as statistically significant.

## Results

**Fig. 1:**
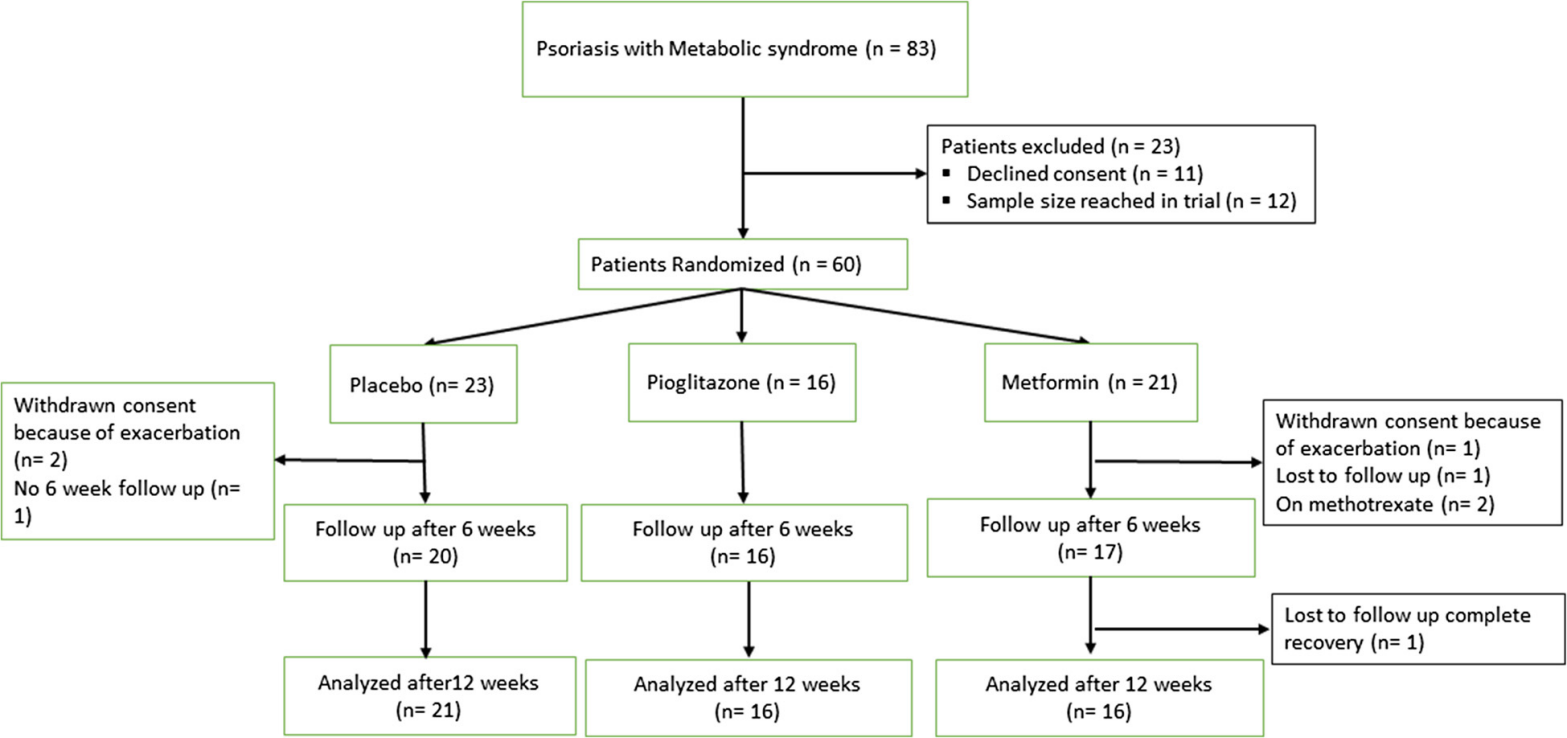
Flowchart of the patients enrolled in the study depicting enrollment, withdrawal and follow up of the subjects

A total of 83 consecutive adult psoriasis patients with MS were screened from June 2010 to April 2011 (Fig. 1). Out of 83 patients, 23 were excluded from the study. 23, 16 and 21 patients were randomized to placebo, pioglitazone and metformin treatment groups respectively. Disposition of patients and reasons for withdrawal were summarized in Fig. 1. Hence, 21 patients in placebo arm, 16 in pioglitazone and 18 patients in metformin arm completed the study. As Intention to treat analysis with last observation carry forward (LOCF) was done, so all the subjects as randomized were included for final analysis.

No significant difference was observed in baseline demographics and MS characteristics among three treatment groups except past history of remission (Table 1).

**Table 1:** Baseline characteristics of three treatment groups

Baseline characteristics	Placebo (*n* = 23)	Metformin (*n* = 21)	Pioglitazone (*n* = 16)	*p*-value
Age (years) Mean (±SD)	46.9 (±10.4)	45.1 (±13.0)	44.0 (±12.9)	0.747
Male/Females, *n* (%)	14/9 (60.9/39.1)	12/9 (57.1/42.9)	9/7 (56.3/43.7)	0.950
Total duration of disease (years) Mean (±SD)	9.1 (±8.6)	6.0 (±6.9)	6.9 (±11.2)	0.492
Seasonal Exacerbation, *n* (%)	13 (56.5)	13 (61.9)	6 (37.5)	0.313
Seasonal improvement, *n* (%)	13 (56.5)	13 (61.9)	5 (31.3)	0.152
Remission, *n* (%)	21 (91.3)	11 (52.4)	10 (62.5)	0.014
Nail involvement, *n* (%)	17 (73.9)	13 (61.9)	12 (75.0)	0.602
Joint involvement, *n* (%)	7 (30.4)	5 (23.8)	4 (25.0)	0.870
DM, *n* (%)	2 (8.7)	3 (14.3)	3 (18.6)	0.653
HTN, *n* (%)	11 (47.8)	10 (47.6)	5 (31.3)	0.523
Family H/O Psoriasis, *n* (%)	4 (17.4)	3 (14.3)	0 (0)	0.225
Alcohol, *n* (%)	6 (26.1)	8 (38.1)	6 (37.5)	0.643
Smoking, *n* (%)	3 (13.0)	3 (14.3)	1 (6.3)	0.727
Vegetarian, *n* (%)	10 (43.8)	11 (52.4)	12 (75.0)	0.144
BMI (kg/m^2^), Mean (±SD)	29.5 (±3.7)	27.6 (±3.7)	27.4 (±4.3)	0.151
Waist Circumference (cm), Mean (±SD)	105.3 (±9.1)	99.0 (±9.9)	100.2 (±8.7)	0.70
ESI, Mean (±SD)	5.9 (±1.6)	5.3 (±1.5)	5.4 (±1.3)	0.412
PGA, Mean (±SD)	3.4 (±0.9)	3.1 (±0.8)	3.2 (±0.8)	0.476
FPG (mg/dl), Mean (±SD)	97.6 (±20.8)	101.9 (±35.1)	103.4 (±28.9)	0.797
Total Cholesterol (mg/dl), Mean (±SD)	184.4 (±37.5)	206.9 (±36.2)	207.2 (±42.3)	0.95
Triglycerides (mg/dl), Mean (±SD)	181.8 (±61.3)	194.3 (±63.1)	200.1 (±55.9)	0.623
HDL (mg/dl), Mean (±SD)	45.1 (±13.5)	44.3 (±6.6)	45.0 (±9.7)	0.968
LDL (mg/dl), Mean (±SD)	107.6 (±35.7)	126.1 (±29.1)	123.1 (±42.3)	0.194
SBP (mmHg), Mean (±SD)	130.4 (±11.5)	130.6 (±12.9)	135.6 (±11.5)	0.344
DBP (mmHg), Mean (±SD)	84.7 (±7.9)	85.9 (±7.9)	85.6 (±8.5)	0.875
Calcium channel blockers, *n* (%)	5 (21.7)	3 (14.3)	3 (18.6)	0.815
Beta blockers, *n* (%)	2 (8.7)	1 (4.8)	0 (0)	0.471
Angiotensin receptor blockers, *n* (%)	2 (8.7)	2 (9.5)	0 (0)	0.456
ACE inhibitors, *n* (%)	1 (4.3)	0 (0)	1 (6.3)	0.543
Diuretics, *n* (%)	0 (0)	0 (0)	1 (6.3)	0.247
Sulfonylureas, *n* (%)	2 (8.7)	0 (0)	1 (6.3)	0.403
Anxiolytics, *n* (%)	1 (4.3)	1 (4.8)	0 (0)	0.684
Lithium, *n* (%)	1 (4.3)	0 (0)	0 (0)	0.441
Antidepressants, *n* (%)	1 (4.3)	2 (9.5)	0 (0)	0.413
Insulin, *n* (%)	0 (0)	1 (4.8)	0 (0)	0.389
Modafinil, *n* (%)	0 (0)	1 (4.8)	0 (0)	0.389
NSAIDS, *n* (%)	0 (0)	1 (4.8)	0 (0)	0.389
Ca, Vitamin D, *n* (%)	0 (0)	1 (4.8)	0 (0)	0.389
Steroids, *n* (%)	0 (0)	0 (0)	1 (6.3)	0.247
Beta 2 agonists, *n* (%)	0 (0)	0 (0)	1 (6.3)	0.247

*DM* diabetes mellitus, *HTN* hypertension, *BMI* body mass index, *ESI* erythema, scaling and Induration, *PFA* physician global assessment, *FPG* fasting plasma glucose, *HDL* high density lipoprotein, *LDL* low density lipoprotein, *SBP* systolic blood pressure, *DBP* diastolic blood pressure, *ACE inhibitors* angiotensin converting enzyme inhibitors

Values are presented as Mean (±SD) or *n* (%)

### ESI and PGA scores and parameters of Metabolic Syndrome (MS)

**Fig. 2:**
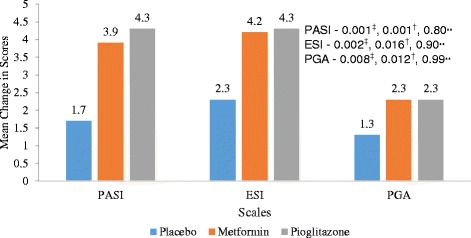
Mean change in PASI, ESI and PGA scores in three treatment groups from baseline (Intention to treat Analysis). || = Inter-group comparisons for PASI, ESI and PGA scores at 12 weeks as compared to baseline was carried out by One Way ANOVA, post hoc test used Scheffe; † = metformin vs placebo, ‡ = Pioglitazone vs placebo,** = metformin vs pioglitazone. PASI - Psoriasis area and severity index, ESI – Erythema, Scaling and Induration, PGA – Physician Global Assessmenty

**Fig. 3:**
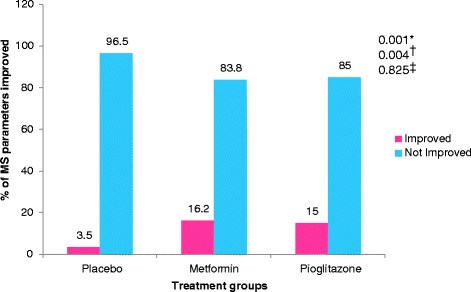
Percentage of parameters of metabolic syndrome (MS) improved following 12 weeks of treatment in placebo, metformin and pioglitazone groups from baseline (Intention to treat Analysis). Inter-group comparisons for percentage of parameters of metabolic syndrome improved carried out by Chi-square test. * = Placebo vs metformin, † = placebo vs pioglitazone, ‡ = metformin vs pioglitazone; MS = Metabolic syndrome

**Fig. 4:**
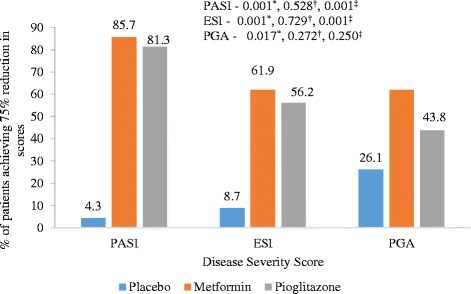
Percentage of patients achieving 75 % reduction in PASI, ESI and PGA scores in placebo, metformin and pioglitazone groups from baseline (Intention to treat Analysis). Inter-group comparisons for 75 % reduction in PASI, ESI and PGA scores between three treatment groups carried out by Chi-square test. * = placebo vs metformin, † = metformin vs pioglitazone, ‡ = placebo vs pioglitazone. PASI - Psoriasis area and severity index, ESI – Erythema, Scaling and Induration, PGA – Physician Global Assessment

**Fig. 5:**
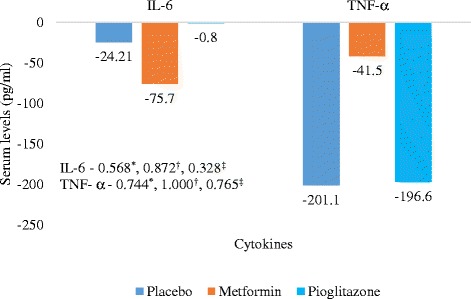
Mean decrease in levels of IL-6 and TNF-α in three treatment groups from baseline in subgroup of patients (Intention to treat Analysis). Values are expressed as Mean ± SD. Inter-group comparisons for IL-6 and TNF-α carried out by One way ANOVA, *- Metformin vs placebo, †- pioglitazone vs placebo, ‡ - metformin vs pioglitazone, IL-6 – Interleukin-6, TNF-α – Tumor necrosis factor-α

Statistically significant improvement was observed in PASI, ESI and PGA scores in pioglitazone (*P* values – PASI = 0.001, ESI = 0.002, PGA = 0.008) and metformin groups (*P* values – PASI = 0.001, ESI = 0.016, PGA = 0.012) as compared to placebo (Fig. 2). There was statistically significant difference in percentage of parameters of MS improved following 12 weeks of treatment in pioglitazone (15 %) and metformin (16.2 %) groups as compared to placebo (3.5 %) (Fig. 3). Statistically significant difference in percentage of patients achieving 75 % reduction in PASI and ESI scores in metformin (*p* value – PASI = 0.001, ESI = 0.001) and pioglitazone groups (*p* value – PASI = 0.001, ESI = 0.001) (Fig. 4). Statistically significant improvement is observed in FPG, total cholesterol and triglycerides levels (Table 2) in metformin and pioglitazone arms as compared to placebo. Significant improvement was also observed in percentage of patients achieving 75 % reduction in PGA scores (Fig. 4) and change in weight and waist circumference in metformin group as compared to placebo (Table 2). Significant improvement was observed in weight, BMI, waist circumference, FPG, triglycerides and total cholesterol after treatment with metformin (Table 2). Similarly improvement was seen in FPG, triglyceride levels, systolic blood pressure (SBP), diastolic blood pressure (DBP), total cholesterol and LDL cholesterol levels after treatment with pioglitazone for 12 weeks (Table 2). No significant change in the IL-6 and TNF-α levels among three groups (Fig. 5).

**Table 2:** Mean Change in individual parameters of metabolic syndrome after 12 weeks of treatment in three treatment groups from baseline (Intention to treat Analysis)

Treatment	Placebo (*n* = 23)		Metformin (*n* = 21)		Pioglitazone (*n* = 16)		ANOVA with post hoc Tukey’s ^b^
Parameters	Mean change [Mean ± SD]	*p* value^a^	Mean change [Mean ± SD]	*p* value^a^	Mean change [Mean ± SD]	*p* value^a^	Between groups, df = 2, *p* value
Weight (kg)	−0.6 ± 3.1	0.338	1.1 ± 1.9	0.016^f^	−0.4 ± 1.7	0.324	0.048^c^, 0.970^d^, 0.129^e^
BMI (kg/m^2^)	−0.1 ± 1.4	0.663	0.4 ± 0.7	0.016^f^	−0.2 ± 0.7	0.370	0.186^c^, 0.995^d^, 0.210^e^
Waist circumference (cm)	−0.9 ± 4.0	0.314	1.9 ± 2.7	0.003^f^	0.9 ± 2.3	0.119	0.013^c^, 0.200^d^, 0.606^e^
FPG (mg/dl)	2.2 ± 10.0	0.312	15.2 ± 19.2	0.002^f^	20.5 ± 17.4	<0.001^f^	0.021^c^, 0.002^d^,0.577^e^
Triglycerides (mg/dl)	1.1 ± 43.3	0.903	44.3 ± 45.4	<0.001^f^	53.3 ± 36.9	<0.001^f^	0.004^c^, 0.001^d^, 0.798^e^
HDL (mg/dl)	−1.7 ± 6.6	0.221	−1.9 ± 4.6	0.060	−1.5 ± 9.6	0.544	0.992^c^, 0.994^d^, 0.974^e^
SBP (mm Hg)	0.0 ± 8.6	1.000	1.7 ± 6.7	0.257	5.1 ± 6.3	0.005^f^	0.725^c^, 0.094^d^, 0.354^e^
DBP (mm Hg)	0.3 ± 7.9	0.876	1.7 ± 4.2	0.077	4.1 ± 5.5	0.009^f^	0.546^c^, 0.085^d^, 0.475^e^
Total Cholesterol (mg/dl)	1.4 ± 29.2	0.816	21.8 ± 25.2	0.001^f^	24.0 ± 29.5	0.005^f^	0.049^c^, 0.042^d^, 0.970^e^
LDL (mg/dl)	−5.9 ± 28.3	0.324	6.6 ± 20.0	0.146	9.8 ± 11.6	0.004^f^	0.151^c^, 0.079^d^, 0.898^e^

*FPG* fasting plasma glucose, *HDL* high density lipoprotein, *SBP* systolic blood pressure, *DBP* diastolic blood pressure, *BMI* body mass index, *LDL* low density lipoprotein

^a^ Intra-group comparisons for weight, BMI, individual parameters of lipid profile and metabolic syndrome carried out by Paired *T*-test

^b^ Inter-group comparisons for individual parameters carried out by One way ANOVA, post hoc Tukey’s test

^c^ Metformin vs placebo

^d^ pioglitazone vs placebo

^e^ metformin vs pioglitazone

^f^ statistically significant difference compared to baseline

No significant difference in the mean number of adverse events in three groups except for weight gain between metformin and pioglitazone (Table 3).

**Table 3:** Adverse events observed during the study in placebo, metformin and pioglitazone treatment groups in topical treatment arm

Adverse Event	Placebo (*N* = 23)	Metformin (*N* = 21)	Pioglitazone (*N* = 16)	*P* value (Fischer’s Exact test)
Redness	1	1	0	>0.99^a^, >0.99^b^,>0.99^c^
Pain	1	0	0	>0.99^a^, >0.99^b^,>0.99^c^
Hyperpigmentation	7	5	4	0.74^a^, >0.99^b^ > 0.99^c^
Hypopigmentation	0	1	1	0.477^a^, 0.41^b^ > 0.99^c^
Exacerbation	2	3	0	0.658^a^, 0.503^b^, 0.243^c^
Hypothyroidism	1	0	0	>0.99^a^, >0.99^b^
Edema	0	0	2	0.162^b^, 0.180^c^
c/o Weight Gain	0	0	2	0.162^b^, 0.180^c^
Anemia	0	0	0	-
Abdominal Pain	0	1	0	0.477^a^, >0.99^c^
Headache	0	0	0	-
Gastritis	0	0	0	-
Nausea	0	0	0	-
Vomiting	0	0	0	-
Dizziness	0	0	0	-
Diarrhea	0	1	0	0.477^a^, >0.99^c^
Heartburn	0	1	0	0.477^a^, >0.99^c^
>3 times SGOT/SGPT	0	0	0	-
Slight increase in SGOT/SGPT	0	0	0	-
Increased TLC	0	0	0	-
Weight gain > 1 kg	8	3	8	0.169^a^, 0.509^b^, 0.030^c^
Recurrence after 3 months	4	6	5	0.377^a^, 0.312^b^, 0.860^c^

Inter group comparison between groups was done by Fischer’s Exact test; *p* – value ≤ 0.05 was considered statistically significant

^a^ placebo vs metformin

^b^ placebo vs pioglitazone

^c^ metformin vs pioglitazone

## Discussion

Baseline characteristics were similar among three treatment groups except for percentage of individuals having remission. The difference observed in baseline characteristic is unlikely to be of clinical significance and could not have accounted for the higher efficacy observed in metformin and pioglitazone groups in comparison to placebo group. All patients were given topical 5 % coal tar treatment. As the compliance achieved is around 90 %, which is ensured by direct questioning and pill count, it is less likely that topical treatment with 5 % coal tar would have resulted in the differences in efficacy among three treatment groups. In our study, metformin and pioglitazone cause significant improvement in PASI, ESI and PGA scores as compared to placebo.

In metformin, pioglitazone and placebo group, 52.4 %, 50 % and 17.4 % of the patients had complete improvement in MS respectively (metformin vs placebo – OR (95 % CI), 5.2 (1.3–20.7), *P* value = 0.019; pioglitazone vs placebo – (OR (95 % CI) = 4.8 (1.1–20.4), *P* value = 0.036; metformin vs pioglitazone – (OR (95 % CI) = 0.9 (0.2–3.3), *P* value = 0.886). Therefore, metformin and pioglitazone beneficial effect on MS parameters might be accounted for the improved efficacy in psoriasis disease itself.

Clinical studies had also demonstrated the proof of efficacy of TZDs in psoriasis. Pioglitazone had demonstrated superior efficacy to placebo group alone as well as in combination therapy with acitretin in psoriasis patients [14, 15]. Two open label studies [16, 17] had demonstrated marked improvement in psoriasis lesions with troglitazone in chronic plaque type psoriasis patients. Robertshaw and Friedman [18] have also demonstrated excellent improvement with pioglitazone in 4 out of 5 patients with chronic plaque type psoriasis in an open label, pilot study.

Study done by Bongartz et al (2005) with pioglitazone 60 mg/day for 12 weeks in psoriatic arthritis patients with tender and swollen joints, demonstrated 60 % of the patients met the psoriatic arthritis response criterion. Mean percentage reduction in PASI was 38 %, along with median tender joint count decreased from 12–4 and median swollen joint count from 5 to 2 (*P* <0.05 for both) [25]. The observed higher percentage reduction in ESI and PGA scores in our study is thus expected and is a demonstration of pioglitazone efficacy.

To the best of our knowledge, effect of metformin in psoriasis patients with MS as done in our study has not been explored earlier. Our study has demonstrated improved efficacy of metformin in psoriasis disease itself as well as features of MS. A population based case control study Brauchli et al estimated the decreased risk of developing a first-time psoriasis diagnosis with metformin use as compared to matched controls [OR = 0.77 (95 % CI – 0.62–0.96)] [26]. One case of psoriasiform drug eruption associated with metformin usage has been observed, which on dechallenge and rechallenege leads to improvement and reappearance of lesions respectively [27]. In view of the case report, it has to be kept in mind that there can be clinical worsening of psoriasis with the use of metformin for treatment of psoriasis.

The anti-proliferative [16, 28], pro-differentiating [29], anti-inflammatory [11, 12, 30] and anti-angiogenic [31, 32] effects of TZDs seen in other studies may underlie the observed beneficial anti-psoriatic effects of pioglitazone. Metformin act through activation of adenosine monophosphate-activated protein kinase (AMPK) in extracellular signal-related kinase (ERK1/2) signaling pathway leading to cell cycle arrest and therefore inhibition of cell proliferation, hallmark of psoriasis [33]. AMPK activation not only inhibits iNOS, dendritic, T cell and monocyte/macrophage activation but also activates IL-10 and TGF-β, thereby exerting its anti-inflammatory action [34]. The anti-proliferative and anti-inflammatory action of metformin might have resulted in improvement of psoriasis.

In diabetics, metformin had shown to decrease HbA_1C_, total and LDL cholesterol, serum triglycerides, fasting insulin levels and improves HDL cholesterol [35–37]. Metformin has shown to improve cardiovascular outcomes by mitigating apoptosis [38]. These results are similar to the results observed in our study. In our study, although an increase in mean weight and BMI was observed with pioglitazone but decrease in mean waist circumference was also observed. Clinical studies had shown despite the weight gain reported in all the studies, which may equal as much as 0.5 kg per month for monotherapy, mean waist to hip ratio remains invariably unchanged. In one study, favorable effect on body fat distribution was demonstrated when patients with type II diabetes were treated with pioglitazone 45 mg/day for 16 weeks [39]. MR imaging revealed that pioglitazone decreased visceral fat area and increased subcutaneous fat mass. With 1H-MR spectroscopy in same group of patients, significant decrease in liver fat content was demonstrated. Thus, thiazolidinedione’s (TZDs) have favorable effects on body fat distribution, intra hepatic fat content and adipose tissue metabolism, all resulting in increased insulin sensitivity.

Our study had revealed a mean fall of 20.5 mg/dl in FPG in psoriasis patients with MS with pioglitazone 30 mg/day. PROFIT-J study have shown improvement in glycemic control, DBP and lipid profile [40]. In clinical trials, pioglitazone monotherapy at a dose of 30 mg/day revealed a dose dependent lowering of FPG by 1.0–3.1 mmol/L and HbA_1C_ reductions ranging between 0.3 and 1.08 % from baseline, significant when compared with placebo [41–45]. Pioglitazone increases peripheral insulin sensitivity, enhancing both splanchnic and peripheral glucose uptake, in patients with type II diabetes in randomized, placebo controlled, 12–26 weeks trial [40, 46, 47]. We observed a mean decrease in serum triglycerides, total cholesterol and LDL cholesterol of 53.3, 24 and 9.8 mg/dl respectively, with pioglitazone 30 mg/day. Lipid profiles generally improved in pioglitazone recipients in three placebo controlled 12–26 week trial [41, 42, 48]. Favorable increases in HDL cholesterol were greater in pioglitazone than placebo recipients in three trials [40, 42, 48].

Mean reduction in SBP and DBP of 5.1 and 4.1 mmHg respectively was measured in pioglitazone group in our study, which was significant as compared to baseline. In a review article by Giles et al, the observed magnitude of reduction was 4–5 mmHg in SBP and 2–4 mmHg in DBP, which were sufficient to significantly reduce cardiovascular event rates [49]. The decrease in cardiovascular risk factors namely lipid profile and blood pressure in our study might contribute to the overall decrease in diabetes and cardiovascular mortality in psoriasis patients with MS.

Increased risk of bladder cancer with the long term use of pioglitazone as shown in French cohort study [50], UK nested case control study [51] and interim results of longitudinal study [52]. However, the 10-year final analysis of longitudinal study [52] did not show any statistically significant findings of increased risk of bladder cancer with long term use of pioglitazone [53]. Similarly, no statistically significant association was found in two Taiwanese studies [54, 55]. Infact, TZDs have shown to decrease the risk of breast, brain, colorectal, ear-nose-throat, kidney, liver, lung, lymphatic, prostate, stomach and uterus cancer significantly [56]. FDA although issued a safety warning, has not withdrawn the drug.

There are no significant differences in metformin as compared to pioglitazone with regard to improvement in psoriasis and MS parameters. But there is significant reduction in weight with the use of metformin and due to controversy of increased risk of bladder cancer associated with pioglitazone; metformin can be preferred over pioglitazone in psoriasis patients with MS.

In subgroup analysis, 10 % of patients in placebo, 14.3 % in metformin and 37.5 % of patients in pioglitazone subgroup had no decline or rather increase in the levels of IL-6 and TNF-α, consistent with the relapse of psoriasis in these patients in next 6 months. Remitting relapsing nature of the disease might be accounted for no significant change in the IL-6 and TNF-α level.

Randomization and placebo control are the strengths of our study. The study also has some limitations. Intermediate dose of pioglitazone (30 mg/day) and metformin (1000 mg/day) was used in the study. Secondly, it was an open label study, although blinded end point assessment was done.

## Conclusion

Insulin sensitizers have shown improvement in the parameters of MS as well as psoriasis disease. With further evaluation in clinical studies, Insulin sensitizers can be used for the management of psoriasis patients with MS.

## Abbreviations

ACE inhibitors: Angiotensin Converting Enzyme Inhibitors
ALT: Alanine Transaminase
AMPK: Adenosine Monophosphate-activated Protein Kinase
ANOVA: Analysis of Variance
AST: Aspartate Transaminase
BMI: Body Mass Index
CVS: Cardiovascular
DBP: Diastolic Blood Pressure
DM: Diabetes Mellitus
ERK1/2: Extracellular Signal-related Kinase 1/2
ESI: Erythema, Scaling and Induration
FPG: Fasting Plasma Glucose
HDL: High density lipoprotein
HTN: Hypertension
IL: Interleukin
LDL: Low Density Lipoprotein
LOCF: Last Observation Carry Forward
MS: Metabolic Syndrome
NCEP ATP III: National Cholesterol Education Program’s Adult Treatment Panel III
OD: Once Daily
PASI: Psoriasis Area and Severity Index
PFA: Physician Global Assessment
PPAR-γ: Peroxisome Proliferator-activated receptor- γ
SBP: Systolic Blood Pressure
SD: Standard Deviation
TNF-α: Tumor Necrosis Factor- α
TZD: Thiazolidinedione
